# Gaps in dengue fever knowledge, attitudes, and practices among healthcare professionals in southeastern Iran

**DOI:** 10.1371/journal.pntd.0013929

**Published:** 2026-02-10

**Authors:** Madineh Abassi, Sara Pourrazavi, Mahmood Moosazadeh, Ahmadali Enayati, Sadigh ahmadzadeh, Gholam-Reza Mehralinasab, Fatemeh Normandipour, Parniya Abolghaseminejad, Somayeh Azimi, Saideh Yousefi

**Affiliations:** 1 Infectious and Tropical Diseases Research Center, Sina Hospital, Tabriz University of Medical Sciences, Tabriz, Iran; 2 Research Center of Psychiatry and Behavioral Sciences, Tabriz University of Medical Sciences, Tabriz, Iran; 3 NonCommunicable Disease Institute, Gastro intestinal Cancer Research Center, Mazandaran University of Medical Sciences, Sari, Iran; 4 Department of Medical Entomology and Vector Control, School of Public Health and Health Sciences Research Center, Mazandaran University of Medical Sciences, Sari, Iran; 5 Department of Disease Prevention and Control, Health Deputy, Jiroft University of Medical Sciences, Jiroft, Iran; 6 Department of Disease Prevention and Control, Health Deputy, Rafsanjan University of Medical Sciences, Rafsanjan, Iran; 7 Student Research Committee, Sirjan School of Medical Sciences, Sirjan, Iran; 8 Department of Health Education and Promotion, School of Public Health, Tehran University of Medical sciences, Tehran, Iran; 9 Social Determinants of Health Research Center, Faculty of Health and Nutrition Sciences, Yasuj University of Medical Sciences, Yasuj, Iran; 10 Department of Public Health, Sirjan School of Medical Sciences, Sirjan, Iran; University of California Irvine, UNITED STATES OF AMERICA

## Abstract

**Background:**

Dengue fever (DF) is one of the most important arboviral diseases and causes significant morbidity and mortality worldwide. Healthcare professionals (HCps) play a crucial role in the prevention, diagnosis, and control of DF. This study aims to assess the knowledge, attitude, and practices (KAP) of HCPs regarding DF management in Kerman Province, southeastern Iran.

**Materials and methods:**

This cross-sectional analytical study was conducted from April to August 2024 in Kerman Province, southeastern Iran. Healthcare professionals (HCPs) from different occupational categories participated. Data were collected using a Persian online questionnaire (www.porsline.ir) covering demographic characteristics and KAP related to DF. The survey link was distributed via social media, email, and official channels. Data were analyzed using SPSS version 24, applying descriptive statistics and multivariable logistic regression, with statistical significance set at α = 0.05.

**Results:**

A total of 307 HCPs participated, with most being female (73.9%) and aged 30–49 years (66.1%). Overall, 30.9% of participants demonstrated a high level of knowledge, with the highest proportion observed among central-level health professionals (41.4%). Additionally, 66.1% and 92.2% of participants scored favorable attitude and practices toward DF, respectively. Multivariable analysis showed that central-level HCPs had significantly higher odds of having good knowledge compared to peripheral staff (AOR: 2.12, 95% CI: 1.06–4.25, P = 0.03).

**Conclusion:**

This study revealed moderate knowledge but generally positive attitudes and strong preventive practices among HCPs in Kerman Province. Significant gaps were identified in transmission and vector control as well as inappropriate prescribing knowledge, underscoring the need for continuous medical education and dissemination of updated treatment guidelines. While central-level staff demonstrated higher knowledge, peripheral-level staff and specialists showed notable deficiencies, highlighting the importance of tailored training initiatives. Strengthening diagnostic confidence, community engagement skills, and evidence-based clinical management will be essential to enhance preparedness and ensure effective dengue prevention and control in this high‑risk region.

## Author summary

Dengue fever (DF) is a major public health threat transmitted by infected *Aedes* mosquitoes, particularly affecting tropical and subtropical regions. This study assessed the knowledge, attitudes, and practices (KAP) of healthcare professionals (HCPs) in Kerman Province, southeastern Iran, regarding DF management. Conducted from April to August 2024, the research involved 307 HCPs and revealed critical gaps in their understanding of DF symptoms, transmission, and prevention strategies. While only 30.9% of participants demonstrated high knowledge, attitudes were generally positive and preventive practices were strong (>90%). However, inappropriate prescribing practices, such as corticosteroid and aspirin use, highlight the need for continuous medical education. General practitioners and central-level HCPs showed better attitudes than specialists and peripheral staff, reflecting differences in roles and responsibilities. These findings underscore the urgent need for structured and targeted training programs to strengthen knowledge, ensure evidence-based clinical management, and enhance community engagement. By addressing these gaps, healthcare systems can improve preparedness and response capacity for potential DF outbreaks in vulnerable regions.

## Introduction

Dengue fever (DF) is an arboviral infection transmitted to humans through the bites of infected invasive *Aedes* mosquitoes, posing a significant global public health threat [1,2]. The disease burden is particularly high in tropical and subtropical regions of Southeast Asia, the Eastern Mediterranean, Central and South America, Africa, and Western Pacific, with Asia reporting the highest incidence among all continents [1–3]. Over the past few decades, DF cases have increased more than 30-fold, resulting in an approximately 100–400 million infections and approximately 25,000–40,000 deaths annually [4]. Projections suggest that by 2080, nearly 60% of the world’s population will be at risk of DF due to the geographic expansion of vectors influenced by climate change [5].

The etiological agent of DF is primarily transmitted by *Aedes aegypti* and *Aedes albopictus*, with clinical manifestations ranging from mild febrile illness to severe forms such as dengue hemorrhagic fever (DHF) and dengue shock syndrome (DSS) [1,2].

In Iran, invasive *Aedes* mosquito species have been reported in both southern and northern regions [6–11]. The first local transmission outbreaks occurred in 2024 in Sistan and Baluchestan and Hormozgon Provinces and have continued to date, both of which border the current study area [12]. More recently, in September 2025, *Aedes aegypti* was detected in Jiroft County, Kerman Province. Given that the southern and southeastern regions of Kerman Province are located near the major DF transmission foci, and considering the high volume of travel and trade connections, it is expected that infected cases may be introduced into Kerman Province [13].

In response, the Iranian Ministry of Health has implemented a DF surveillance program, organizing workshops, seminars, and training sessions at national, provincial, and county levels. The scope and quality of these educational activities largely depend on the presence of invasive *Aedes* species and local transmission in each region. The primary objective of these programs is to strengthen the knowledge, attitudes, and practices (KAP) of society, particularly healthcare professionals (HCPs).

Understanding DF symptoms, vector ecology, transmission routes, and prevention and control strategies is essential for HCPs in areas such as Kerman Province, which have not yet reported local transmission. Timely diagnosis and preventive counseling for imported cases can reduce the risk of viral reservoirs spreading to regions where invasive species are present, thereby preventing potential outbreaks in Iran. One of the major challenges in DF prevention and control is the insufficient KAP among communities, especially HCPs [14–16]. Since healthcare workers play a vital role in educating the public, preventing transmission, and managing cases, their high level of KAP is critical for reducing outbreaks [17,18].

Previous KAP studies have been conducted in northern and northwestern Iran, which are geographically and culturally distinct from southeastern Iran and distant from DF foci [14,19]. In contrast, a study in Sistan and Baluchestan, the most important DF focus in Iran, revealed that 71% of health personnel had not received any training on DF, which may have contributed to the outbreak in that region [20].

The direct (medical and non-medical) and indirect (disability-adjusted life years, DALYs) costs of DF impose a substantial burden on patients, families, and the government [21]. Therefore, implementing effective prevention programs based on appropriate KAP can significantly reduce disease-related costs. As no study has yet assessed the KAP of DF among HCPs across all universities in Kerman Province, this research was conducted for the first time in this high-risk area to identify gaps in knowledge, attitudes, and practices. The findings will provide guidance for future educational programs on DF prevention and control.

In this study, we aimed to evaluate the KAP of HCPs in Kerman Province regarding DF management. Specifically, we focused on critical domains including symptoms, treatment, diagnostic methods, national care programs, vector management, and personal protective measures. By assessing these domains, we sought to identify existing gaps and strengths in current practices. The results will highlight areas requiring improvement and provide policymakers with actionable insights tailored to the specific needs of each domain and target group. Ultimately, effective implementation of these findings can enhance the capacity for timely and appropriate responses in the event of a DF outbreak or vector introduction.

## Materials and methods

### Ethics statement

The study was approved by the ethical committee of Sirjan School of Medical Sciences (IR.SlRUMS.REC.1402.022). Informed written consent was obtained from all the participants before filling out the questionnaire. Those not willing to participate were given the right to do so. Confidentiality of responses was also ensured throughout the research process.

### Study design and population

**Fig 1:**
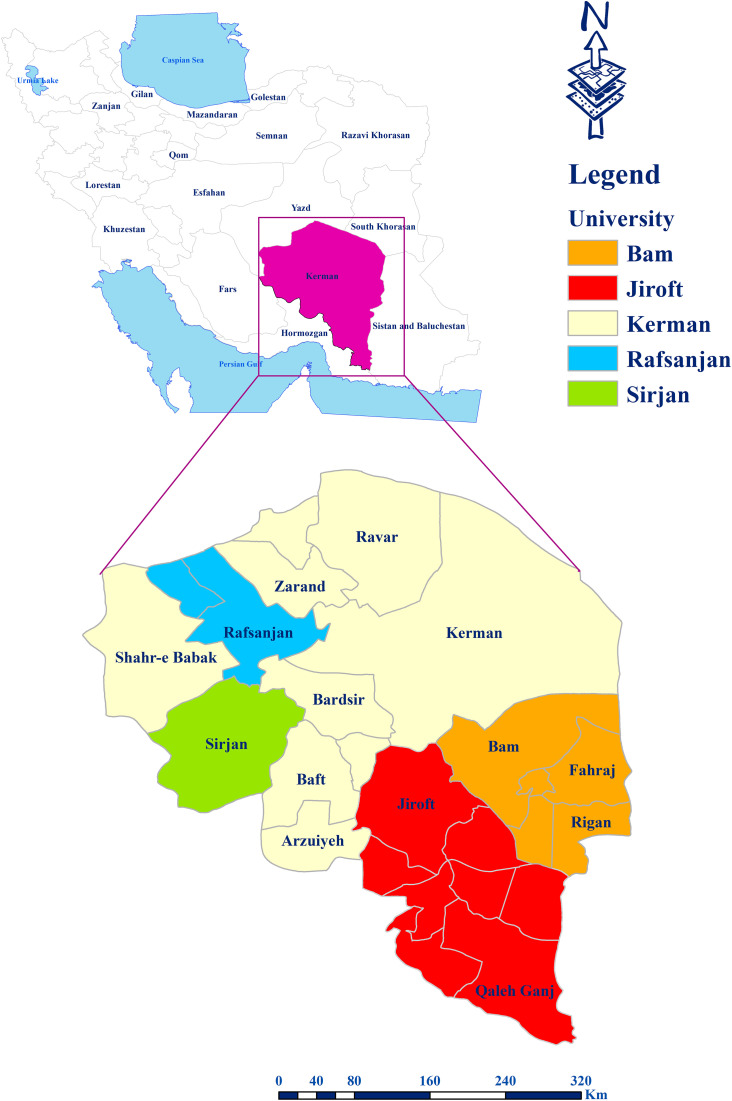
Counties of Kerman Province by coverage of the five universities of Medical Sciences. Basemap: OpenStreetMap contributors; data available under ODbL (https://www.openstreetmap.org).

This cross-sectional study was conducted between April and August 2024 in Kerman Province, southeastern Iran. The province covers more than 11% of Iran’s area and shares borders with Sistan and Baluchestan, Hormozgan, South Khorasan, Fars, and Yazd Provinces. Kerman includes 25 counties, with healthcare services divided among five universities of medical sciences: Kerman (9 counties), Jiroft (8 counties), Bam (5 counties), Rafsanjan (2 counties), and Sirjan (1 county) (Fig 1).

The study population consisted of healthcare professionals (HCPs) responsible for timely diagnosis, control, and management of diseases. Participants were stratified into four predefined occupational categories reflecting their training and functional roles in dengue fever (DF) prevention, control and management:

- **General practitioners (GPs):** Physicians with an MD degree working in primary care clinics, public health centers, and hospitals, responsible for first-contact diagnosis, notification, and initial clinical management.
- **Specialist physicians (SPs):** Board-certified physicians in infectious diseases, emergency medicine, pediatrics, or internal medicine, providing advanced diagnostic and inpatient/outpatient management, and guiding treatment protocols.
- **Central-level healthcare professionals:** Non-physician public health staff (e.g., epidemiologists, environmental health officers, disease control experts; typically BSc/MSc) employed at Health and Treatment Deputies and county health networks, overseeing surveillance, outbreak investigation, vector control planning, and inter-sectoral coordination.
- **Peripheral-level healthcare professionals:** Non-physician frontline staff (e.g., public health nurses, health inspectors, surveillance officers; typically BSc/MSc) working in comprehensive health centers, delivering community education, case referral, vector control implementation, and routine data reporting.

The sample size was calculated using a single population proportion formula:

n = (Zα/2)^2^ × p (1 − p)/ d^2^ assuming a prevalence of adequate DF knowledge at 0.50 (to maximize sample size when prior estimates were unavailable), with a 95% confidence level (*Z*α/2 = 1.96) and 5% margin of error (*d* = 0.05), the required sample size was 384. Due to time and access constraints, 307 HCPs were recruited via convenience sampling across all five universities networks. This shortfall is acknowledged as a limitation, and findings are interpreted as indicative rather than strictly representative of all HCPs in the province.

The Persian online questionnaire was designed on www.porsline.ir and distributed via SMS, email, WhatsApp, Instagram, LinkedIn, and official letters. Participants provided electronic informed consent and a brief study introduction was displayed before sequential items. To prevent duplicate submissions, the platform restricted multiple entries per device via cookies/session controls, and server-side checks flagged identical metadata. No suspected duplicates were retained after data cleaning. Participation was voluntary and independent; no supervisory pressure was applied.

**Inclusion criteria:** currently employed HCPs in Kerman Province within one of the four categories, ability to complete an online survey, and consent to participate.

**Exclusion criteria:** internship-only positions, administrative staff without DF-related duties, and incomplete submissions.

### Measures

A structured questionnaire was used, divided into four sections: demographic, knowledge, attitude, and practice. The instrument’s validity and reliability were previously established (Cronbach’s alpha = 0.79), indicating acceptable internal consistency. The questionnaire was adapted from prior KAP instruments to the DF context and local setting while preserving construct coverage, and has been used in previous studies [14,19,22].

***1) Demographic characteristics***

Variables included age, gender, job group, university of employment, workplace type (private/public), history of DF training, and training source. Training sources encompassed academic workshops and social media platforms (e.g., Telegram, Instagram, WhatsApp), including both general user-generated content and educational material shared by health authorities.

***2) Knowledge***

Comprised 47 questions (symptoms: 14; transmission: 9; clinical management: 3; prevention/vector control: 21) (S1 Table). Each correct answer scored 1; incorrect 0. Negatively keyed items were reverse-scored. A score ≥ 70% (≥ 33 correct responses) indicated high knowledge.

***3) Attitude***

Included 14 Likert-scale items scored from 0 (strongly disagree) to 4 (strongly agree), total range 0–56. Negatively keyed items were reverse-scored. A score ≥ 70% (≥ 39 points) indicated a positive attitude (S2 Table).

***4) Practice***

Comprised 16 items across two epidemiological scenarios (S3 Table).

- Vector un-established (5 items): actions appropriate when dengue vectors are not yet reported nationally (e.g., travel-related risk counseling, early notification of suspected imported cases, general community education).
- Local DF transmission (11 items): actions appropriate when local transmission is present (e.g., case classification and referral, household-level source reduction, coordination with vector control teams, adherence to case management protocols).

Correct responses scored 1; incorrect 0; negatively keyed items were reverse-scored. Scores ≥ 70% (≥11.2 correct answers) were classified as high practice. This differentiation reflects distinct operational guidance before and after vector establishment.

Overall, maximum scores were 47 for knowledge, 56 for attitude, and 16 for practice.

### Statistical analysis

Data were analyzed using IBM SPSS Statistics, version 24. Normality of continuous variables was assessed using the Kolmogorov–Smirnov test. Descriptive statistics (frequencies and percentages) summarized categorical variables. Pearson’s chi-square tested associations between demographic variables and dichotomized KAP outcomes. Multivariable logistic regression estimated adjusted odds ratios (AORs) for predictors of high KAP levels. Statistical significance was set at α = 0.05.

## Results

### Study population demographic characteristics

A total of 307 HCPs participated in this study. The majority (73.9%) were female, and most were aged between 30 and 49 years (66.1%). Peripheral level HCPs represented the largest job group (43.3%). The highest participation rates were from Kerman and Jiroft Universities of Medical Sciences (32.6%). Nearly 94% of participants were employed in the public sector, while only 6% worked in private institutions. Academic training and workshops were the primary sources of dengue fever-related information for 64.9% of participants (Table 1).

**Table 1:** Association between demographic characteristics and knowledge, attitude, and practice (KAP) scores of healthcare professional’s (HCP_S_), Kerman Province, southeastern Iran, 2024.

Demographic characteristics		Knowledge (N. (%))		*p*-value	Attitude (N. (%))		*p*-value	Practice (N. (%))		*p*-value
		Poor	Good		Poor	Good		Poor	Good	
**Gender**	**Female**	157 (51.1)	70 (22.8)	1.000	82 (26.7)	145 (47.2)	0.340	20 (6.5)	207 (67.4)	0.172
	**Male**	55 (17.9)	25 (8.1)		22 (7.2)	58 (18.9)		4 (1.3)	76 (24.8)	
**Age group**	**<30**	62 (20.2)	19 (6.2)	0.398	29 (9.4)	52 (16.9)	0.714	6 (2)	75 (24.4)	0.917
	**30-39**	68 (22.1)	33 (10.7)		37 (12.1)	64 (20.8)		8 (2.6)	93 (30.3)	
	**40-49**	67 (21.8)	35 (11.4)		32 (10.4)	70 (22.8)		9 (2.9)	93 (30.3)	
	**≥50**	15 (4.9)	8 (2.6)		6 (2)	17 (5.5)		1 (0.3)	22 (7.2)	
**Job group**	**Peripheral-level healthcare professionals**	97 (31.6)	36 (11.7)	0.142	51 (16.6)	82 (26.7)	0.006*	9 (2.9)	124 (40.4)	0.177
	**General practitioners (GPs)**	39 (12.7)	13 (4.2)		12 (3.9)	40 (13)		1 (0.3)	51 (16.6)	
	**Specialist physicians (SPs)**	35 (11.4)	17 (5.5)		25 (8.1)	27 (8.8)		6 (2)	46 (15)	
	**Central-level healthcare professionals**	41 (13.4)	29 (9.4)		16 (5.2)	54 (17.6)		8 (2.6)	62 (20.2)	
**University**	**Kerman**	73 (23.8)	27 (8.8)	0.003*	39 (12.7)	61 (19.9)	0.224	9 (2.9)	91 (29.6)	0.879
	**Sirjan**	29 (9.4)	11 (3.6)		15 (4.9)	25 (8.1)		4 (1.3)	36 (11.7)	
	**Jiroft**	71 (23.1)	29 (9.4)		25 (8.1)	75 (24.4)		6 (2)	94 (30.6)	
	**Rafsanjan**	21 (6.8)	25 (8.1)		16 (5.2)	30 (9.8)		4 (1.3)	42 (13.7)	
	**Bam**	18 (5.9)	3 (1)		9 (2.9)	12 (3.9)		1 (0.3)	20 (6.5)	
**Workplace**	**Private**	13 (4.2)	6 (2)	1.000	8 (2.6)	11 (3.6)	0.459	1 (0.3)	18 (5.9)	1.000
	**Public**	199 (64.8)	89 (29.0)		96 (31.3)	192 (62.5)		23 (7.5)	265 (86.3)	
**Information Sources**	**Academic training/Workshops**	115 (41.2)	66 (23.7)	0.183	51 (18.3)	130 (46.6)	0.141	15 (5.4)	166 (59.5)	0.971
	**Social media**	70 (25.1)	28 (10.0)		36 (12.9)	62 (22.2)		8 (2.9)	90 (32.3	

* p < 0.05 considered statistically significant.

### Knowledge

The mean knowledge score was 30.23 ± 4.77 (scale range: 17–44). Overall, 30.9% of participants achieved high knowledge. Regarding specific domains, 60.6% of participants were knowledgeable about symptoms, 39.4% about transmission pathways, 55.7% about clinical management, and only 14.7% about prevention and vector control. These findings highlight that while knowledge of symptoms and clinical management was relatively strong, awareness of transmission and vector control remained limited. Central-level HCPs had the highest proportion of good knowledge (41.4%). Across universities, Rafsanjan showed the highest suitable knowledge (54.3%), while Bam had the lowest (14.3%). knowledge levels varied significantly among five universities (χ² (4) = 15.645, p-value = 0.004) (Table 1).

**Fig 2:**
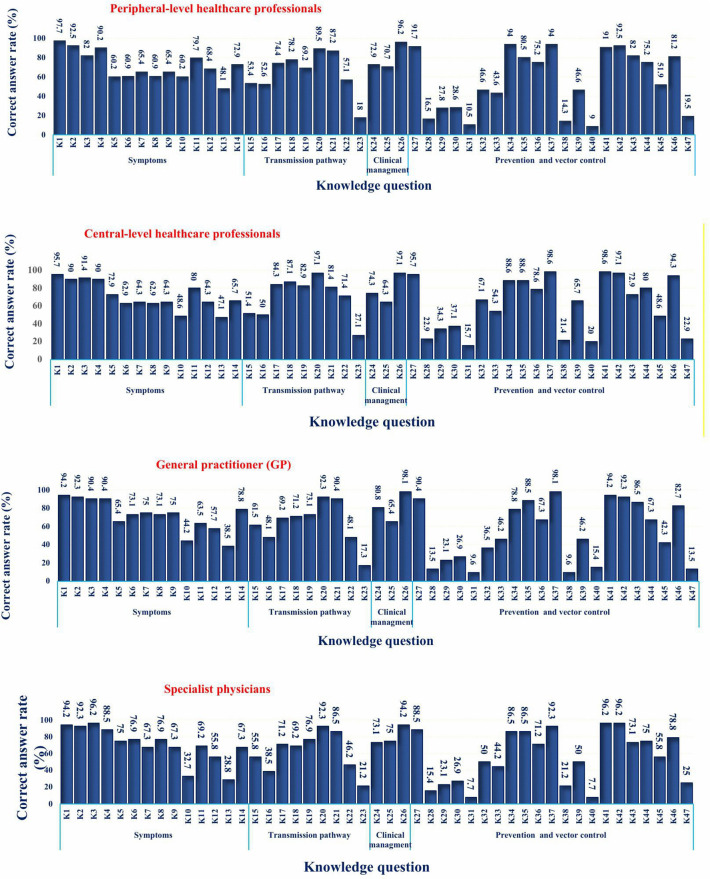
Correct response status to dengue fever knowledge questions among healthcare professional (HCPs) by occupation in Kerman Province, southeastern Iran, 2024. *Note: Detailed survey questions are provided in S1 Table.

Building upon the overall knowledge distribution presented in Table 1, a more detailed item-level analysis revealed both strengths and weaknesses across occupational groups. Knowledge was strong regarding common symptoms such as fever, headache, and joint pain, and most participants correctly identified *Aedes* mosquitoes as the primary vector. However, awareness was limited regarding non-specific symptoms not associated with DF, including cough, chest pain, dizziness, microcephaly, and swelling of hands and feet. Another deficiency was observed in recognizing that flies and ticks do not transmit the disease. Participants also showed insufficient knowledge about the fact that DF is primarily transmitted during the daytime and that urban areas can serve as transmission settings. Across job groups, many participants incorrectly considered unsuitable habitats (e.g., riverbanks, rice fields, animal waste areas) as potential breeding sites. In the domain of prevention, although measures such as window screens and eliminating water-holding containers were well recognized, knowledge was insufficient regarding the importance of environmental management in reducing vector populations. Using a 70% threshold, these domains were classified as inadequate knowledge. Fig 2 illustrates the proportion of correct responses to each item across the total sample, with stratification by occupational group provided for additional detail. The primary aim is to reflect overall response patterns rather than exhaustive group-by-group comparisons.

### Attitude

The overall attitude of participants was positive, with a mean score of 41.79 out of 56. Using a 70% threshold, 66.1% of participants demonstrated a favorable attitude towards DF. General practitioners (76.9%) and central-level HCPs (77.1%) had the highest proportions of individuals with favorable attitudes. A statistically significant difference was observed among occupational groups (p = 0.006), while no significant differences were found across other demographic characteristics (Table 1). These findings indicate that attitudes were generally favorable, though professional background influenced the level of positivity.

**Fig 3:**
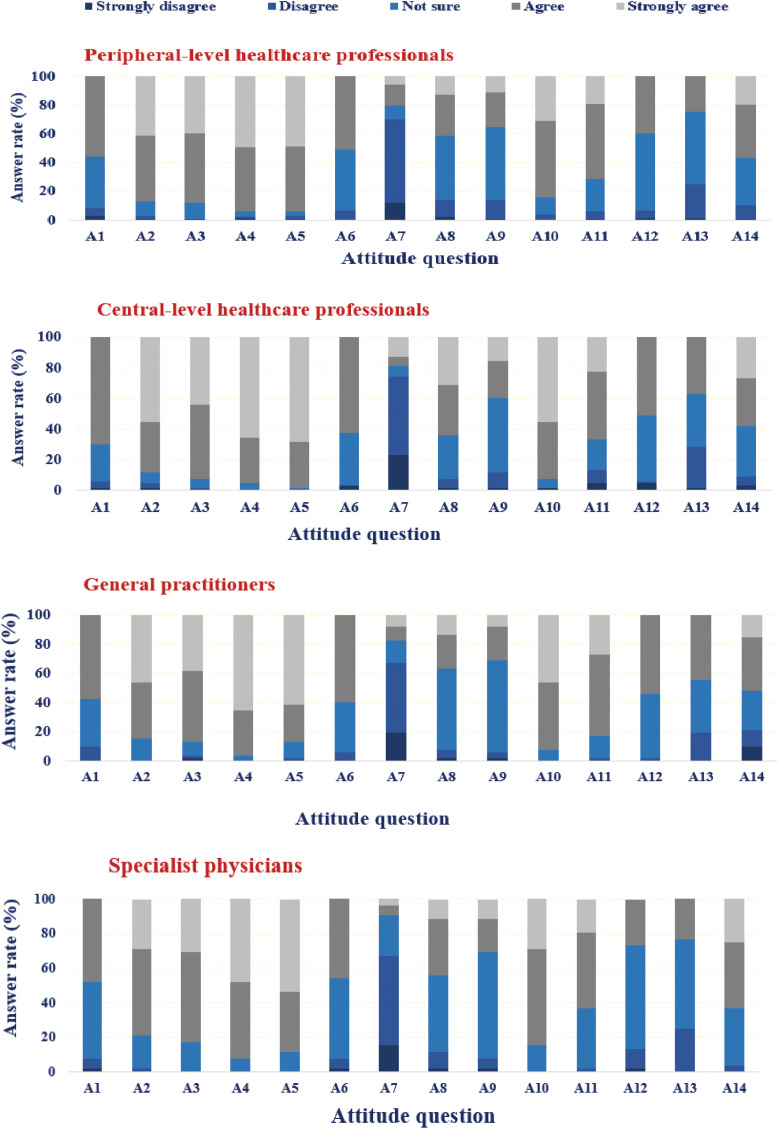
Correct responses to dengue fever attitude questions among healthcare professionals (HCPs) by occupation in Kerman Province, southeastern Iran, 2024. *Note: Detailed survey questions are provided in S2 Table.

Fig 3 presents the distribution of responses to individual attitude items across occupational groups. Attitudes towards dengue were generally favorable, with strong agreement on its danger, preventability, and the importance of community participation in vector control. Most participants correctly disagreed with the statement that only the government is responsible, reflecting awareness of shared responsibility. However, substantial uncertainty remained in diagnostic and clinical management items, where more than 40% of respondents selected ‘Not sure’ or ‘Disagree’. These findings highlight that while general attitudes were positive, gaps remain in diagnostic awareness and clinical management practices, indicating areas for targeted training (Fig 3).

### Practice

**Fig 4:**
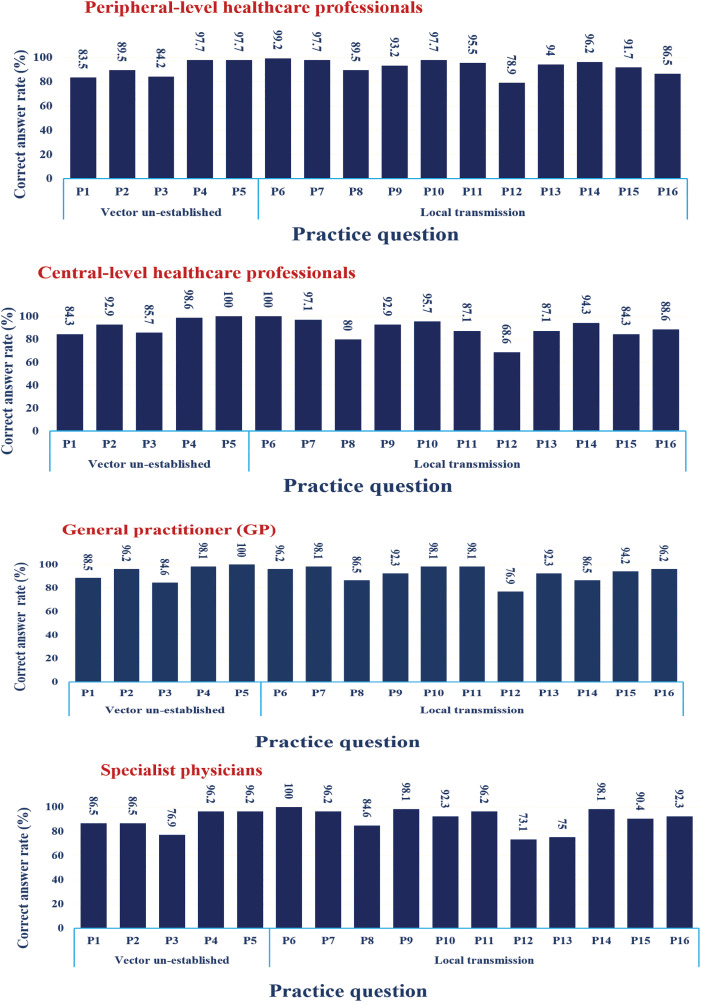
Correct responses to dengue fever practice questions among healthcare professionals (HCPs) by occupation in Kerman Province, southeastern Iran, 2024s. *Note: Detailed survey questions are provided in S3 Table.

Overall, participants reported strong practices concerning dengue fever prevention, with 92.2% responding affirmatively and only 7.8% negatively. Under conditions of vector un-establishment, 88.9% of respondents expressed confidence in their practices, while performance under local transmission conditions was even higher, with 94.5% reporting appropriate actions. Item-level analysis revealed particularly strong practices in healthcare services, immediate case reporting, insecticide spraying, and larval control, all exceeding 95% across job groups. Preventive measures such as window screens, insect repellents, and covering water-storage containers were also widely adopted. However, weaker practices were observed in encouraging public participation (68–79%) and the use of mosquito coils (80–89%), indicating areas requiring further emphasis. Chi-square analysis showed no statistically significant differences in overall practice across job groups or universities. Fig 4 illustrates the proportion of correct responses to each item across the total sample, with stratification by occupational group provided for additional detail.

### Factors associated with KAP level of HCPs towards DF prevention

Univariate and multivariable analyses showed that central-level HCPs had higher odds of good knowledge compared to peripheral staff (OR: 1.90, 95% CI: 1.03–3.50, P = 0.03; AOR: 2.12, 95% CI: 1.06–4.25, P = 0.03). Rafsanjan University participants also demonstrated significantly better knowledge compared to Kerman University (AOR: 3.56, 95% CI: 1.58–8.06, P = 0.002). For attitude, general practitioners had significantly higher odds of favorable attitudes (AOR: 3.31, 95% CI: 1.35–8.12, P = 0.009), and central-level health professionals also showed better attitudes (OR: 2.09, 95% CI: 1.08–4.05, P = 0.02). In contrast, specialists demonstrated lower odds of favorable attitudes (OR: 0.67, 95% CI: 0.35–1.28, P = 0.23). For practice, no statistically significant associations were observed across job groups, workplace, gender, age, or university (Table 2). These findings suggest that professional background and institutional affiliation play an important role in shaping knowledge and attitudes, while practice levels remained consistently high across groups.

**Table 2:** Analysis of predictors for knowledge, attitude, and practice of healthcare professional’s (HCPs), Kerman Province, southeastern Iran, 2024.

Independent variable	Knowledge level		Attitude level		Practice level	
	Univariate OR (95% CI), P-value	Multivariable AOR (95% CI), P-value	Univariate OR (95% CI), P-value	Multivariable AOR (95% CI), P-value	Univariate OR (95% CI), P-value	Multivariable AOR (95% CI), P-value
**Job group**						
**Peripheral-level healthcare professionals (R)**	**1**	**1**	**1**	**1**	**1**	**1**
**General practitioners (GPs)**	**0.89 (0.43-1.87), 0.77**	**1.39 (0.61-3.16), 0.42**	**2.07 (0.99-4.31), 0.051**	**3.31 (1.35-8.12), 0.009**	**3.70 (0.45-29.97), 0.22**	**4.04 (0.46-35.43),0.2**
**Specialists (SPs)**	**1.30 (0.65-2.62), 0.44**	**1.63 (0.69-3.85), 0.26**	**0.67 (0.35-1.28), 0.22**	**1.16 (0.49 -2.75), 0.73**	**0.55 (0.15-1.65), 0.29**	**0.55 (0.15-2.01), 0.37**
**Central-level healthcare professionals**	**1.90 (1.03-3.50), 0.03**	**2.12 (1.06-4.25), 0.03**	**2.09 (1.08-4.05), 0.02**	**1.91 (0.92-3.96), 0.08**	**0.56 (0.20-1.52), 0.25**	**0.47 (0.15-1.42), 0.18**
**Workplace**						
**Private (R)**	**1**	**1**	**1**	**1**	**1**	**1**
**Public**	**0.96 (0.35-2.63), 0.95**	**0.81 (0.26-2.49), 0.72**	**1.45 (0.56-3.73), 0.43**	**1.48 (0.50-4.38), 0.47**	**0.64 (0.08-5.01), 0.67**	**0.65 (0.07-5.44), 0.69**
**Gender**						
**Female (R)**	**1**	**1**	**1**	**1**	**1**	**1**
**Male**	**1.83 (0.60-5.54), 0.28**	**0.93 (0.48-1.79), 0.83**	**1.49 (0.85-2.61), 0.16**	**1.49 (0.75-2.97), 0.25**	**1.01 (0.58-1.76),0.94**	**1.84 (0.55-6.18), 0.32**
**Age group (year)**						
**<30 (R)**	**1**	**1**	**1**	**1**	**1**	**1**
**30-39**	**1.58 (0.81-3.06), 0.17**	**1.62 (0.77-3.39), 0.19**	**0.96 (0.52-1.77), 0.9**	**1.17 (0.58-2.35), 0.66**	**0.93 (0.30-2.79), 0.89**	**0.91 (0.26-3.12), 0.88**
**40-49**	**1.70 (0.88-3.28), 0.11**	**1.55 (0.74-3.26), 0.24**	**1.22 (0.65-2.26), 0.52**	**1.41 (0.69-2.89), 0.34**	**0.82 (0.28-2.42), 0.72**	**0.95 (0.27-3.32), 0.94**
**≥50**	**1.74 (0.64-4.73), 0.27**	**1.46 (0.45-4.71), 0.51**	**1.58 (0.56-4.45), 0.38**	**2.16 (0.59-7.85), 0.24**	**1.76 (0.20-15.41), 0.61**	**2.11 (0.2-21.63), 0.52**
**Information Source**						
**Academic training/Workshops (R)**	**1**	**1**	**1**	**1**	**1**	**1**
**Social media**	**1.43 (0.84-2.44), 0.18**	**1.8 (0.99-3.24), 0.051**	**1.48 (0.87-2.49), 0.14**	**1.78 (1-3.18), 0.04**	**0.98 (0.40-2.40), 0.97**	**1.09 (0.42-2.83), 0.85**
**University**						
**Kerman (R)**	**1**	**1**	**1**	**1**	**1**	**1**
**Sirjan**	**1.02 (0.45-2.33), 0.95**	**1.37 (0.54-3.46), 0.5**	**1.06 (0.5-2.26), 0.86**	**0.88 (0.34-2.30), 0.8**	**0.89 (0.25-3.07), 0.85**	**0.81 (0.18-3.47), 0.77**
**Jiroft**	**1.1 (0.59-2.04), 0.75**	**0.94 (0.47-1.87), 0.86**	**1.91 (1.04-3.51), 0.03**	**1.41 (0.7-2.86), 0.32**	**1.54 (0.53-4.52), 0.42**	**1.66 (0.51-5.39), 0.39**
**Rafsanjan**	**3.21 (1.55-6.67), 0.002**	**3.56 (1.58-8.06), 0.002**	**1.19 (0.57-2.48), 0.62**	**1.61 (0.7-3.69), 0.26**	**1.03 (0.30-3.56), 0.95**	**1.38 (0.38-5.03), 0.61**
**Bam**	**0.45 (0.12-1.65), 0.22**	**0.53(0.13-2.11), 0.36**	**0.85 (0.32-2.21), 0.74**	**0.98 (0.33-2.90), 0.97**	**1.97 (0.23-16.51), 0.52**	**2.09 (0.22-19.54), 0.51**

## Discussion

Ensuring adequate KAP among HCPs during epidemics such as DF is critical for effective disease management. Strengthening KAP not only enhances outbreak preparedness and resilience but also reduces unnecessary costs and improves disease outcomes [14,16,19,23]. Since 2024, southern and southeastern Iran have increasingly faced DF outbreaks, with expanding endemic foci and the progressive spread of *Aedes* vectors into new urban areas. The recent detection of *Ae. aegypti* in Kerman Province, bordering endemic regions, underscores the imminent risk of early dengue outbreak in this province and highlights the urgent need for preparedness and preventive strategies [13,24].

In this study, HCPs demonstrated a moderate level of knowledge of DF, with only 30.9% achieving high scores. In contrast, attitudes were generally positive, as 66.1% of participants met the threshold for favorable perspectives, and practices were particularly strong, with over 92% reporting appropriate preventive actions. These findings suggest that while knowledge remains limited, especially in certain domains, positive attitudes and robust preventive practices provide a strong basis for strengthening DF control efforts.

Multiple studies have confirmed moderate levels of knowledge among healthcare professionals regarding DF, with significant variations across regions and professional groups. Yusuf et al., 2019 reported that nearly half (49.3%) of HCPs demonstrated a moderate level of knowledge.Similarly, Abbasi et al., 2024 found that 80.4% of HCPs had inadequate knowledge about DF. Other studies support these findings: Kantha et al., 2016 noted that 58% of staff nurses possessed a moderate level of knowledge, while Hoang Thi Nam Giang et al., 2021 found significant knowledge gaps, with over 80% of physicians and nurses lacking prior dengue infection training. These studies, covering multiple countries and healthcare settings, consistently highlight the need for enhanced dengue fever education and training among healthcare professionals [14,25–28].

In this study, significant variation in knowledge levels was observed across the five universities of medical sciences in Kerman Province, with Rafsanjan reporting the highest suitable knowledge and Bam the lowest. These disparities likely reflect differences in institutional resources and training opportunities related to vector-borne disease programs. The recent detection of *Ae. aegypti* in Jiroft University in September 2025 further underscores the urgency of strengthening preparedness [13], as this area may become the first site of dengue outbreaks in the province. Given the study province’s geographic position, bordering endemic regions such as Sistan and Baluchestan and Hormozgan, the risk of rapid spread is considerable. Therefore, all universities must implement structured and targeted capacity-building programs for their HCPs to ensure timely recognition, diagnosis, and control of DF before local transmission becomes established.

In our survey, social media referred broadly to platforms such as Telegram, Instagram, and WhatsApp, encompassing both general user-generated content and educational material shared by health authorities and professionals. Reliance on these sources was not associated with improved knowledge or attitude. While social media can facilitate rapid information exchange and professional communication, its heterogeneous nature—including both evidence-based content and misinformation—may explain the lack of significant association observed in our study [29–33].

General practitioners, as the first point of contact in the healthcare system, play a critical role in the early diagnosis and notification of DF [34]. In our study, they demonstrated favorable attitudes, reflecting their awareness of the importance of timely recognition and reporting. Their position in primary care settings allows them to influence both clinical outcomes and public health surveillance [27,35]. However, gaps in knowledge highlight the need for continuous training to ensure that GPs can accurately differentiate dengue from other febrile illnesses and avoid inappropriate management during the early stages of care.

Specialist physicians, including in infectious diseases, pediatrics, and internal medicine, are responsible for advanced diagnostic and inpatient management. Interestingly, our findings indicated that specialists had lower odds of favorable attitudes compared to other groups. This may reflect their narrower focus on hospital-based management rather than preventive or community-level interventions [36]. While their expertise is essential for severe and complicated cases, strengthening their engagement in preventive strategies and community awareness could enhance the overall effectiveness of dengue control [37].

Central-level HCPs demonstrated the highest proportion of good knowledge, which can be explained by their responsibilities in surveillance, outbreak investigation, and guideline dissemination. Their role places them at the core of planning and coordination, ensuring that technical information is translated into operational strategies. In contrast, peripheral-level HCPs, who are directly engaged in community education and vector control implementation, exhibited relatively weaker knowledge. This gap is concerning, as frontline staff are essential for mobilizing communities and sustaining preventive measures. Tailored educational interventions are therefore needed to strengthen practical knowledge among peripheral staff, while emphasizing diagnostic confidence and preventive attitudes among physicians and central-level HCPs.

In this study, HCPs demonstrated significant knowledge asymmetry regarding DF. While recognition of symptoms (60.6%) and clinical management (55.7%) was relatively strong, awareness of transmission pathways (39.4%) and vector control (14.7%) remained critically limited. This difference underscores the urgent need for targeted educational interventions that move beyond symptom-focused awareness to comprehensively address vector ecology, transmission dynamics, and prevention strategies. Limited recognition of atypical symptoms increases the risk of misdiagnosis, particularly in regions where malaria and typhoid are prevalent, highlighting the importance of enhanced diagnostic training and integration of rapid tests [38,39].

Most HCPs (77.5%) correctly identified the primary vector of DF, the *Aedes* mosquito, consistent with other studies conducted in Iran, Pakistan, and Bhutan [14,40–42]. Knowledge gaps were most evident in transmission and vector ecology, with misconceptions about habitats and biting behavior of *Aedes* mosquitoes. Similar findings from Cameroon and Pakistan confirm that HCPs often underestimate the role of urban breeding sites [29,42–46]. Since vector control remains the cornerstone of dengue prevention in the absence of curative treatment, these deficiencies pose serious challenges. Evidence from Thailand and systematic reviews demonstrates that integrated vector management—source reduction, environmental sanitation, and community participation—is both cost-effective and essential for outbreak prevention [47–49].

Although clinical management knowledge was moderate, inappropriate prescribing practices remain a concern [50,51]. Nearly 40% of respondents reported corticosteroid use, despite WHO guidelines contraindicating their use due to risks of gastrointestinal bleeding and stress ulceration. 25.4% would also prescribe aspirin to control fever in DF patients, with no apparent regard to the anti-thrombotic activities of this drug, which would lead to decreased platelet function and subsequently massive hemorrhaging [23]. Similar misuse has been reported in Ethiopia and Bangladesh, emphasizing the need for continuous medical education and dissemination of updated treatment protocols [52–54].

Attitudes towards dengue were generally favorable, with strong agreement on its danger, preventability, and the importance of community participation [55,56]. Most participants rejected the notion that only the government is responsible, reflecting an awareness of shared responsibility. However, diagnostic uncertainty persisted, with over 40% expressing doubt in clinical management items, a gap that requires targeted training in laboratory diagnostics (ELISA, PCR) and standardized guidelines.

Practices were consistently strong, particularly in case reporting, insecticide spraying, and larval control (>95%), and preventive measures such as window screens and covering water-storage containers were widely adopted. Yet weaker practices were observed in encouraging public participation (68–79%) and reliance on mosquito coils (80–89%). Sustained vector control depends on active community engagement rather than short-term chemical interventions, as shown in Thailand and Latin America where community-based source reduction programs proved more cost-effective and sustainable [57–59]. These findings highlight the need to strengthen communication skills and participatory approaches among healthcare professionals to ensure long-term resilience against dengue outbreaks.

Although some survey items were relatively simple, they were designed to capture baseline knowledge in a setting where dengue fever is newly emerging. The results emphasize the urgent need for continuous, evidence‑based training programs to build sustainable capacity among healthcare professionals and ensure long‑term resilience against dengue outbreaks.

In summary, this study revealed critical gaps in dengue-related knowledge, attitudes, and practices among healthcare professionals in southeastern Iran. While practices were generally strong, limited awareness of transmission dynamics, vector ecology, and appropriate clinical management highlights the urgent need for continuous, evidence-based training. Addressing these gaps through structured educational programs and strengthening community engagement will be essential for sustainable outbreak preparedness.

### Limitations

This study has several limitations. First, it relied on self‑reported data from healthcare professionals, which may not fully reflect actual compliance with therapeutic guidelines. Second, although the calculated sample size was 384, only 307 participants could be recruited due to time and access constraints. This shortfall, combined with the use of convenience sampling, may have introduced selection bias and limits the representativeness and generalizability of the findings. In particular, uneven distribution across occupational groups and institutions could have influenced observed differences. Third, participants may have accessed educational information from sources beyond those identified in this research, which could affect reported knowledge and practices. Finally, the lower attitude scores observed among specialist physicians may partly reflect sampling bias, as specialists were primarily recruited from hospital settings and may not represent all specialists in the province. These limitations should be considered when interpreting the results.

## Conclusions

The rising global burden of dengue fever and the expanding habitat of *Aedes* mosquitoes due to climate change underscore the importance of preparing healthcare professionals for potential outbreaks. This study in Kerman Province, a non‑endemic region of Iran, identified substantial gaps in knowledge, attitudes, and practices, emphasizing the need for targeted educational interventions. Central‑level health professionals demonstrated stronger preparedness compared to peripheral staff, reflecting the benefits of structured training in centralized settings. In contrast, workplace type, age, and reliance on social media were not associated with improved KAP outcomes. To enhance outbreak readiness, integrating dengue‑focused training into continuous medical education particularly for peripheral healthcare professionals is essential. Future research should evaluate the long‑term impact of such programs and explore innovative approaches to strengthen sustainable capacity building.

## Supporting information

S1 Table Knowledge questions about dengue fever in Kerman Province, southeastern Iran (N = 307). (DOCX)

S2 Table Attitude questions about dengue fever in Kerman Province, southeastern Iran (N = 307). (DOCX)

S3 Table Practice questions about dengue fever in Kerman Province, southeastern Iran (N = 307). (DOCX)
