# Supplementary material for: Gaps in dengue fever knowledge, attitudes, and practices among healthcare professionals in southeastern Iran
Source: PLoS Negl Trop Dis. 2026 Feb 10;20(2):e0013929. doi: 10.1371/journal.pntd.0013929 (PMC12919928; doi:10.1371/journal.pntd.0013929)
Supplement: S1 Table — (DOCX) [file pntd.0013929.s001.docx]

**S1. Table** Knowledge questions about dengue fever in Kerman Province, southeastern Iran. (N=307)

| Question | | | Number |
| --- | --- | --- | --- |
| Symptoms | | Is fever one of the symptoms of dengue? | K1 |
|  |  | Is headache one of the symptoms of dengue? | K2 |
|  |  | Is joint pain one of the symptoms of dengue? | K3 |
|  |  | Is muscle pain one of the symptoms of dengue? | K4 |
|  |  | Is eye pain one of the symptoms of dengue? | K5 |
|  |  | Are skin rashes one of the symptoms of dengue? | K6 |
|  |  | Is stomach pain one of the symptoms of dengue? | K7 |
|  |  | Is diarrhea one of the symptoms of dengue? | K8 |
|  |  | Is cough one of the symptoms of dengue? | K9 |
|  |  | Is chest pain one of the symptoms of dengue? | K10 |
|  |  | Is dizziness one of the symptoms of dengue? | K11 |
|  |  | Is microcephaly considered a symptom of dengue? | K12 |
|  |  | Are swelling of hands and feet and joint inflammations prominent symptoms of dengue? | K13 |
|  |  | Is conjunctivitis a prominent symptom of dengue? | K14 |
| Transmission routes | | Are flies vectors of dengue? | K15 |
|  |  | Are ticks vectors of dengue? | K16 |
|  |  | Do malaria vectors (Anopheles) transmit dengue? | K17 |
|  |  | Are Aedes mosquitoes vectors of dengue? | K18 |
|  |  | Does dengue transmit by sex? | K19 |
|  |  | Does person-to-person contact transmit dengue? | K20 |
|  |  | Does blood transfusion transmit dengue? | K21 |
|  |  | Is dengue a(n) Most Urban/ Most Rural disease? | K22 |
|  |  | Dengue transmission by vectors mostly occur? At night/ During the daytime/ Both day and night | K23 |
| Clinical management | | Do you prescribe aspirin for dengue? | K24 |
|  |  | Do you prescribe corticosteroids for dengue? | K25 |
|  |  | Is dengue a reportable disease? | K26 |
| Prevention and vector control | **Vector breeding places** | Human-made containers (buckets, used tires, drinking water barrels) | K27 |
|  |  | Riverbanks | K28 |
|  |  | Wide drainage systems | K39 |
|  |  | Rice fields | K30 |
|  |  | Stagnant dirty water | K31 |
|  |  | Animal feces | K32 |
|  |  | Pastures and stables | K33 |
|  | **Personal prevention** | Is there a vaccine available for preventing dengue? | K34 |
|  |  | Insect repellents | K35 |
|  |  | Long-sleeved white shirts and pants | K36 |
|  |  | Window screens | K37 |
|  |  | Personal hygiene measures | K38 |
|  |  | Daily vitamin C intake | K39 |
|  |  | Sleeping under bed nets at night | K40 |
|  | **Vector control method** | Eliminating or emptying water-holding containers | K41 |
|  |  | Covering household water-storage containers | K42 |
|  |  | Using chemical agents (larvicides) in water-holding containers | K43 |
|  |  | Managing solid waste | K44 |
|  |  | Regularly cutting grass | K45 |
|  |  | Storing used tires under a roof or keeping them protected from rain with appropriate covers. | K46 |
|  |  | Water management in rice fields | K47 |
