# Supplementary material for: Gaps in dengue fever knowledge, attitudes, and practices among healthcare professionals in southeastern Iran
Source: PLoS Negl Trop Dis. 2026 Feb 10;20(2):e0013929. doi: 10.1371/journal.pntd.0013929 (PMC12919928; doi:10.1371/journal.pntd.0013929)
Supplement: S2 Table — (DOCX) [file pntd.0013929.s002.docx]

**S1. Table 2.** Attitude questions about dengue fever in Kerman Province, southeastern Iran. (N=307)

| Number | Question |
| --- | --- |
| A1 | Dengue is a dangerous disease. |
| A2 | Iran is at risk of dengue vector invasion. |
| A3 | Dengue is preventable. |
| A4 | Control of dengue breeding places is a strategy to prevent this disease |
| A5 | Tires, containers, and pots around homes are suitable breeding sites for dengue vectors. |
| A6 | People must actively participate in controlling dengue vectors. |
| A7 | Only the government is responsible for controlling dengue vectors. |
| A8 | PCR methods are used to confirm dengue? |
| A9 | ELISA methods are used to confirm dengue? |
| A10 | Reporting cases of dengue is a national priority. |
| A11 | Dengue is treatable. |
| A12 | Follow-up visits for suspected dengue patients are necessary. |
| A13 | It is necessary to perform complete blood counts at least every 48 hours in suspected dengue patients. |
| A14 | Suspected dengue patients with warning signs and no access to complete blood counts should not start fluid therapy. |
