# Supplementary material for: Gaps in dengue fever knowledge, attitudes, and practices among healthcare professionals in southeastern Iran
Source: PLoS Negl Trop Dis. 2026 Feb 10;20(2):e0013929. doi: 10.1371/journal.pntd.0013929 (PMC12919928; doi:10.1371/journal.pntd.0013929)
Supplement: S3 Table — (DOCX) [file pntd.0013929.s003.docx]

**S1. Table 3.** Practice questions about dengue fever in Kerman Province, southeastern Iran. (N=307)

| Number | Question | |
| --- | --- | --- |
| P1 | Larval surveys at entry points | **Operations in *Aedes* un-established** |
| P2 | Installation of ovitraps at entry points |  |
| P3 | Entomological Surveillance throughout the country |  |
| P4 | Healthcare services |  |
| P5 | Immediate reporting of suspected cases of the disease |  |
| P6 | Insecticide spraying | **Operations in local transmission** |
| P7 | Larval control with insecticides |  |
| P8 | Use of mosquito coils to reduce vector populations |  |
| P9 | Installation of screens on windows to reduce vector populations |  |
| P10 | Use of fans to reduce vector populations |  |
| P11 | Sleeping under mosquito nets at night |  |
| P12 | Encouraging public participation to reduce vector breeding sites |  |
| P13 | Elimination of small water containers around homes to reduce vector populations |  |
| P14 | Trimming grass around homes to reduce vector populations |  |
| P15 | Use of insect repellents |  |
| P16 | Covering household water-storage containers |  |
